# The Effects of Artificial Night Lighting on Tail Regeneration and Prey Consumption in a Nocturnal Salamander (*Plethodon cinereus*) and on the Behavior of Fruit Fly Prey (*Drosophila virilis*)

**DOI:** 10.3390/ani12162105

**Published:** 2022-08-17

**Authors:** Sharon E. Wise, Alex Rohacek, Ashley E. Scanlon, Tiffany Cabrera, Bryant W. Buchanan

**Affiliations:** Department of Biology, Utica University, Utica, NY 13502, USA

**Keywords:** artificial light at night (ALAN), dim nocturnal illumination, light pollution, caudal regeneration, fruit fly activity, insect, predation, amphibian, plethodontid salamander

## Abstract

**Simple Summary:**

Amphibians, including salamanders, are declining worldwide at an alarming rate due to a variety of factors that include habitat decline and destruction and environmental pollutants. Artificial light at night (ALAN) due to human activities is a nearly ubiquitous pollutant and can have serious consequences for amphibians. We examined the impact of ecologically-relevant levels of ALAN on tail regeneration in the eastern red-backed salamander, prey consumption by these salamanders and behavior of their fruit fly prey. We found that ALAN reduced the rate of salamander tail regeneration at some light levels above the naturally dark nocturnal illumination and increased the activity of their prey but not always in a simple, linear fashion. Thus, ALAN, even at very low levels, can influence the physiology and regeneration of a nocturnal salamander.

**Abstract:**

As human development continues to encroach into natural habitats, artificial light at night (ALAN) has increasingly become a concern for wildlife. Nocturnal animals are especially vulnerable to ALAN, as the physiology and behavior of nocturnal species have evolved under conditions associated with predictably dark environments. Studies exposing amphibians to constant bright light provide evidence for changes to normal metabolism, growth, and behavior, but few of these studies have used treatments of dim ALAN comparable to that found in affected habitats. Eastern red-backed salamanders, *Plethodon cinereus,* use their tails for fat storage and communication, are capable of tail autotomy as an antipredator mechanism, and can regenerate the tail in its entirety. We examined the effect of different, ecologically-relevant intensities of ALAN on the rate of tail regeneration in adult *P. cinereus*. We hypothesized that ALAN would increase tail regeneration rates such that salamanders exposed to higher levels of light at night would regenerate tails faster than those exposed to lower light levels. In a controlled laboratory setting, we exposed salamanders (N = 76) in test chambers to nocturnal illuminations of 0.0001 lx (no ALAN, natural nocturnal illumination dark control), 0.01 lx (weak ALAN), 1 lx (moderate ALAN), or 100 lx (bright ALAN, equal to dim daytime and our day lighting treatment) for a period of 90 d immediately following tail autotomy. In addition, because these salamanders eat mostly live, moving prey, we investigated the impact of ALAN on the behavior of prey (*Drosophila virilis*) fed to the salamanders in our laboratory trials, which could alter feeding and regeneration rates in salamanders. We predicted that prey consumption would not be affected by ALAN and measured both prey consumption and prey behavior (activity) to examine the potential influence on regeneration. For tail regeneration, we found a non-monotonic response to ALAN, with salamanders exposed to nocturnal illuminations 0.1 lx and 100 lx regenerating tails significantly slower than salamanders in the 0.0001 lx or 1 lx treatments. Prey consumption did not differ among light treatments; however, fruit fly activity increased with increasing ALAN. These results suggest that ALAN influences regeneration rates, but the rate of regeneration is not dose-dependent and is not explained easily by prey consumption or movement of prey. We suggest that tail regeneration in these salamanders may involve a complex mechanism of altered gene expression and/or modulation of hormonal activity (corticosterone, melatonin, serotonin, and/or prolactin) at different intensities of nocturnal lighting.

## 1. Introduction

Increased human development has resulted in the introduction of artificial light at night (ALAN) into natural habitats around the globe [1]. This artificial light at night, i.e., light pollution, is a major ecological problem that seems to have a widespread, negative impact on many different species [2,3,4,5,6,7]. Although it is clear that wildlife living in urban areas is exposed to a greater intensity of ALAN from point (direct) sources, such as street lighting (10 lx), ambient sources—particularly sky glow—can also produce artificial light levels of up to 1 lx (comparable to dawn/dusk twilight light intensity) [2] in areas surrounding urban centers far from point sources, such that very few areas in the world are unaffected by ALAN [1]. The global exposure to ALAN may have consequences for biodiversity world-wide [8]; however, there are relatively few studies that examine the effect of ecologically-relevant dim light at night (dALAN) on the ecology, behavior, and physiology of organisms; thus, controlled experiments examining the consequences of dALAN, such as that associated with sky glow are necessary and important [9]. Additionally, studies examining dose responses to ALAN are even scarcer and are essential in determining the threshold levels of ALAN that affect organisms [10]. Most studies of the effects of nocturnal ALAN on amphibians have used dichotomous treatments of nocturnal illuminations that are either very (or even completely) dark or brightly lit, thereby missing the opportunity to assay the effects of intermediate, ecologically relevant amounts of ALAN [11,12]. Thus, studies of the effects of ecologically-relevant dALAN on amphibian biology are essential and necessary for understanding how light pollution affects these animals [10,13].

Dim light intensities, representative of light levels associated with changing lighting at dawn or dusk can be important zeitgebers that synchronize circadian cycles and regulate photoperiodic behavior due to different gene expression in different neurons, e.g., morning (M) and evening (E) oscillators in *Drosophila* [14,15,16,17] and so it is reasonable to predict that biological systems might respond differently to dALAN than to darker or brighter ALAN. Additionally, ALAN intensity diminishes as the inverse of the squared distance from the light source and so animals in habitats adjacent to sources of lighting would experience different amounts of ALAN (from bright to exceedingly dim) depending on their distance from the source. Thus, in order to understand the effects of ALAN on organisms living in affected habitats, it is important to recognize that those organisms may not respond identically throughout the habitat if light intensities vary.

Nocturnal species may be particularly susceptible to ALAN since they are active under normally dark conditions and have evolved physiological functions and behavior associated with dark ambient illumination [3]. Many species of amphibians are nocturnal, and their numbers have been declining globally over the last century due to environmental perturbations, such as habitat destruction, climate change, environmental pollution, and invasive species [18,19,20,21]. Although multiple drivers are probably responsible for global amphibian decline [20,21,22,23,24], local population-level declines are likely due to a more specific set of stressors unique to that habitat [21,22]. ALAN has the potential to be a nearly ubiquitous stressor for nocturnal amphibians [8], but it is one of the least studied forms of pollution in amphibians, with surprisingly few studies examining its effects [13]. ALAN can affect behavior, reproductive cycles, hormones, and metabolic rates of amphibians through altered photoperiodism and vision [11,12].

Salamanders are important components of many forest and aquatic ecosystems and are sensitive to environmental perturbations; thus, they are excellent indicators of the health of some ecosystems [25,26]. The eastern red-backed salamander, *Plethodon cinereus*, is a major predator of invertebrates in deciduous forests of eastern North America [27], occurring at extremely high densities in some locations [28], such that the biomass of these salamanders may be higher than that of any other vertebrates in some forests [29,30]. The behavior and ecology of these nocturnal salamanders have been extensively studied [31], making them a model species for studies of the effects of light pollution on an ecologically important nocturnal animal.

*Plethodon cinereus*, similar to many other species of salamanders, can autotomize and completely regenerate its tail [32]. Tail autotomy in this and similar species may serve as a predatory defense mechanism allowing the animal to escape capture by sacrificing its tail [33,34]; and tail elongation following autotomy is an energetic priority for salamanders [35]. However, tail loss may put a salamander at a disadvantage because the tail is important for fat storage during winter brumation [36]; tail loss reduces reproductive output, with fewer ova produced following regeneration [37]; the tail is involved in territorial communication and defense, including scent marking [38], threat displays [39,40], and fighting [41]; and subsequent escape from predation is reduced, while the tail is regenerating. Thus, factors influencing regeneration rates may, in turn, affect territoriality, communication, spatial dynamics, reproduction, and even survival of individuals within populations.

Although the effect of ALAN on tail regeneration has not previously been studied in salamanders, constant and relatively high levels of light at night enhanced tail regeneration in a gekkonid lizard (*Hemidactylus flaviviridis*) and limb regeneration in eastern red-spotted newts (*Notophthalmus viridescens*), whereas constant darkness or pinealectomy slowed regeneration [42,43,44]. This indicates an inhibition of melatonin production [45] that is responsible for increased rates of regeneration, perhaps because (1) DNA synthesis and mitotic activity fluctuate on a daily serotonin–melatonin rhythm regulated by light [46,47], with melatonin slowing mitotic activity [48,49,50] and (2) production of prolactin is increased, stimulating regeneration [51,52]. Conversely, ALAN may decrease the rate of tail regeneration by increasing corticosterone levels as a stress response, as has been demonstrated in tadpoles of *Bufo valliceps* and *Rana berlandieri* [53]. Elevated levels of corticosterone reduced rates of regeneration in the plethodontid salamander, *Desmognathus ochrophaeus* [54].

ALAN may also affect prey capture and consumption, which are necessary to provide energy and nutrients for tail regeneration [55], by altering vision or activity. The eastern red-backed salamander, similar to many other species of salamanders, is nocturnally active and negatively phototactic, such that these animals emerge from the leaf litter and from under cover objects (rocks and logs) to forage at night [12,56] and avoid brightly lit areas [57,58], such as those that might be associated with ALAN. Therefore, if ALAN is sufficient to trigger photonegative behaviors, salamanders may be more likely to express escape behaviors and may be less apt to forage in brightly lit conditions. Additionally, visual cues are important to foraging *P. cinereus* and under some conditions, e.g., when olfactory cues were not available, individuals of *P. cinereus* oriented toward prey sooner at higher nocturnal illuminations (unpublished data cited in [12,59]). Because these salamanders orient toward and use vision to detect and capture moving prey, if ALAN alters the activity of insect prey, it may indirectly impact tail regeneration rates in salamanders by influencing prey detection and capture. In our study, we fed salamanders fruit flies, *Drosophila virilis*. Although literature examining the effect of dALAN on the activity of *D. virilis* is not available, the activity of wild-type *Drosophila (Sophophora) melanogaster* was greater when exposed to dALAN (0.03 lx) than when exposed to complete darkness [60]. Wild-type *D. (S.) melanogaster* have distinctly bimodal morning and evening activity under laboratory conditions, whereas *D. virilis,* displayed 96% of their locomotor activity during daylight, and although seemingly crepuscular, may show more activity closer to dusk [61,62,63]. It is possible that the addition of night lighting may increase the nocturnal activity level of *D. virilis* in our study thereby directly or indirectly influencing the foraging behavior and tail regeneration rates of the salamanders. Therefore, we examined the nocturnal behavior of prey as part of this study.

We hypothesized that an increase in the nocturnal ambient illumination equivalent to ecologically-relevant levels of light pollution would alter the rate of tail regeneration in *P. cinereus*, although we did not predict a direction because there are multiple physiological and behavioral factors that may influence tail regeneration as previously discussed. Furthermore, because the rate of regeneration (tail growth) may be influenced by food consumption and prey behavior, we hypothesized that consumption of prey items (*D. virilis)* would be positively correlated with the size of the salamander but would not be influenced by different nocturnal lighting treatments or behavior of the prey, as there may be opposing effects of light at night on successful prey capture (i.e., increased illumination may increase prey capture efficiency but avoidance of lighted areas by the salamanders may reduce time spent foraging). We also hypothesized that ALAN would increase the nocturnal behavior (activity) of *D. virilis* over what would be exhibited under natural dark illuminations.

## 2. Materials and Methods

### 2.1. Collection and Maintenance of Animals

Adult male and female salamanders of *P. cinereus* (N = 76) were collected from under rocks and logs in a forested area in Frankfort, Oneida County, NY, USA from 6 August−10 September 2010. Salamanders were maintained in individual Petri dishes (14.5 cm diameter × 1.5 cm deep) lined with moistened filter paper. Prior to experimentation, the animals were maintained at 18 °C with 80% relative humidity on a 12L:12D photoperiod (100 lx day: 0.0001 lx night). The salamanders were fed a diet of fruit flies (*D. virilis*) *ad libitum* with monthly vitamin and calcium supplementation.

Fruit flies in both experiments were bred and maintained on *Drosophila* media (Ward’s Science, Rochester, NY, USA) supplemented with yeast. Flies were maintained on a 12L:12D photoperiod (100 lx day: 0.0001 lx night, same as the dark control lighting treatment for salamanders) for at least 8 d before the study began and for the duration of the experiment.

### 2.2. Light Chambers

**Figure 1:**
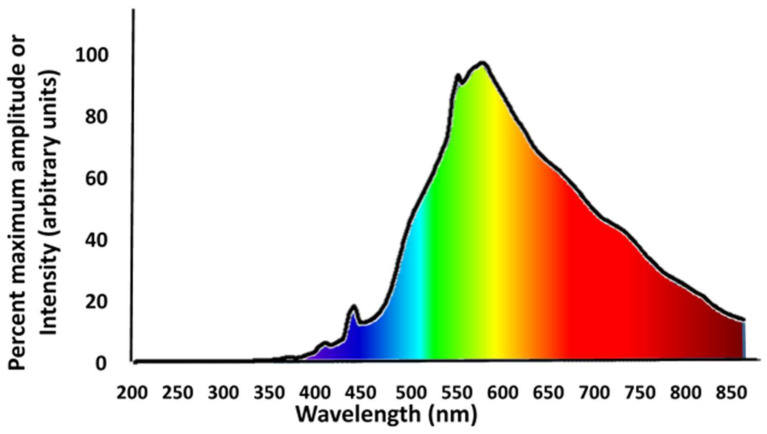
Spectral distribution of light from one representative LED lamp used to produce day- and night-lighting in our experiments measured with a StellarNet EPP2000C reflectance spectrometer.

We used 16 light chambers constructed using black metal cabinets (46 × 61 × 76 cm). Day lighting (100 lx) was provided by white CK-1 LED lights (Connex, Massapequa, New York, NY, USA) in 10 light strips with 4 LEDs per strip (representative spectrum in Figure 1) masked with metal tape to create daytime illumination of 100 lx at the location of the salamander in the chamber. The photoperiod was set at 12L:12D. Another single LED lamp (light strip with 4 of the same white LEDs) provided night lighting, and it was mounted in the center of the field of other LEDs and then masked using metal tape to create the needed nocturnal treatment illumination. All salamanders in all treatments were exposed to the same day-time light intensity of 100 lx in every chamber. We established four different nocturnal lighting treatments (100 lx, 1 lx, 0.01 lx, and 0.0001 lx) with lighting chambers randomized for each lighting treatment. There were four replicate chambers for each lighting treatment (four replicate chambers per treatment × four lighting treatments = 16 chambers total). All chambers were arrayed in the same room and lighting treatments were randomly assigned to chambers within the array of chambers. The 0.0001 lx nocturnal light treatment served as the natural, dark control treatment, simulating a clear, starry night without moonlight. The experimental lighting treatments corresponded to low intensity of light pollution (0.01 lx, equivalent to full moonlight), moderate light pollution (1 lx, equivalent to dawn/dusk), and constant daylighting (100 lx; equivalent to dim day light illuminations in the forest, to abolish photoperiodicity in that treatment). A white mat was placed on the floor of each chamber to reflect light from the LED lamps for more even ambient lighting. Each chamber was outfitted with an infrared camera and infrared light source (>850 nm) (amphibian visual and circadian systems are not impacted by infrared light [64,65]), which were used only for the fruit fly nocturnal activity experiment. Temperature and relative humidity were monitored in each chamber throughout the experiment (19.2–26.6 °C; 15–72%) but did not differ between chambers by more than 1 °C and 5% relative humidity at any given time. Although temperature may influence rates of tail regeneration in salamanders (for another plethodontid, *D. conanti*) [55], the difference in temperature was not associated in any way with specific light treatments, rather the temperature was fairly even across all chambers but varied over time due to changes in the heating and cooling system of the building that affected all chambers equally; the largest variation in temperature occurred from the activation of the heating system in the building during the first 30 d of the experiment.

All light levels were measured using an IL1700 Research Radiometer (International Light, Peabody, MA, USA) with SHD033 detector and ZCIE scotopic and Y photopic light filters to measure illuminations (lx) at a 45-degree angle relative to the substrate of a salamander’s container at the center of each chamber. Radiometric measurements (W/cm^2^) were also taken but not presented in this study. Spectra of the LED light sources were obtained using an EPP2000C reflectance spectrometer (StellarNet, Tampa, FL, USA) with a range of 200–850 nm with the infrared lights associated with the infrared cameras off (a representative spectrum is presented in Figure 1). The lightguide for the reflectance spectrometer was held at a 45-degree angle to a 50 mm RS50 white halon 97% reflectance standard disc placed in the center of each chamber.

### 2.3. Growth and Tail Regeneration in Red-Backed Salamanders

Prior to the start of the experiment, all salamanders (N = 76) were photographed, weighed (g), and measured for snout-vent length (SVL in mm, from the tip of snout to the posterior end of cloaca) and tail length (from the posterior end of the cloaca to the tip of tail) as in [66]. SVL did not differ among lighting treatments (F = 1.941; df = 3, 72; *p* = 0.131). Sex was identified by shining light from a fiber optic light through the body cavity and looking for testes or eggs. Tail autotomy was induced by giving a slight pinch to the tail at the appropriate position along the length of the tail with thin forceps and holding with gentle pressure until the salamander autotomized its tail (as in [41]). These salamanders have a specialized wound healing mechanism; the salamander constricts its muscles and blood vessels just anterior to the point of pressure from the forceps, detaching the tail between the vertebra with minimal to no loss of blood; the skin detaches posterior to the site of the muscle constriction and then closes over the tail wound [33]. Salamanders are commonly found in nature with missing and regenerating tails (missing as much as 61% [67]). Therefore, our laboratory procedure did not stress salamanders more than what they would periodically experience in their natural habitat. Autotomy was induced at a point along the tail to result in an autotomized tail length that was approximately 15% of the SVL. After tail autotomy, salamanders were placed into fresh Petri dishes and then randomly assigned to one of four replicate chambers in one of the four nocturnal lighting treatments: 0.0001 lx, 0.01 lx, 1 lx, and 100 lx (N = 19 for each lighting treatment).

While in the light chambers, all salamanders were fed a diet of fruit flies (*D. virilis*) ad libitum. SVL and regenerated tail lengths of the salamanders were remeasured at 30, 60, and 90 d after tail autotomy during daylight hours.

To determine whether light treatment affected tail regeneration over time, we compared the length of regenerated tails at 30, 60, and 90 d for salamanders exposed to the four different nocturnal light treatments using repeated measures MANCOVA (IBM SPSS V 28). Nocturnal light treatments (4 nocturnal light intensities) and sex (2: male and female) were used as main effects and light by sex as the interaction effect, with regeneration lengths (mm) at 30, 60, and 90 d as the response variables. We also used body condition at the start of the experiment as a covariate to remove the effects of differences in massiveness or fatness on tail regrowth at the start of the experiment. Body condition was measured using the residuals of empty-stomach mass in g, regressed on SVL in mm [68], as in other studies using condition in salamanders [69,70,71]. Salamanders with larger positive residuals were in better condition (more massive for a given snout-vent length) than those with larger negative residuals. We performed post-hoc ANCOVAs when the main effects from the ANCOVA were significant. Following significant results with ANCOVA, pair-wise comparisons were performed using LSD tests. We used two-tailed tests with α = 0.05. Because body size influenced regeneration rates in the plethodontid salamander *Desmognathus quadramaculatus* (although they used both sexually mature and immature individuals and did not determine sex or body condition as we did) [72], we also performed the same analyses with SVL or SVL and body condition as covariates. The results for all analyses resulted in the same significant effects of light treatments on regeneration at 60 and 90 d and so are not presented in the Results section.

### 2.4. Consumption of Prey by Red-Backed Salamanders

To determine whether salamanders differed in the consumption of prey based on sex and light treatment, we monitored the number of prey consumed by salamanders following the 90-day-trial, using the same salamanders as in the 90-day-regeneration study. We assumed that fruit fly consumption for this 10-day-period would provide an estimate of the relative number of flies consumed by those same salamanders during our 90-day-testing period because the number of prey eaten by individuals of *P. cinereus* is highly repeatable [73]. We started this test 30 d following the 90-day-experiment. Individual salamanders remained in the same light chamber under the same light treatment for the entire 120 d.

Seven days before experimentation on fruit fly consumption, flies were removed from the Petri dishes and salamanders were not fed, allowing the salamanders to clear food from their guts, which was confirmed by viewing the salamander’s gastrointestinal tract by shining a fiber optic lamp through the body cavity. Then, we collected and counted flies by briefly anesthetizing them using CO_2_ gas. Initially, all salamanders were fed 30 fruit flies (*D. virilis*). The salamanders were fed by pouring the separated flies into each salamander’s Petri dish. Each day during photophase, the number of flies remaining in the Petri dish was counted. If the number of living flies remaining in the Petri dish was less than 10, then the remaining flies were removed and 30 new flies were added; if more than 10 flies remained, new flies were not added. This procedure ensured that each salamander would always have an adequate number of flies in its visual field should it choose to feed, i.e., reducing the effect of changes in prey density on feeding rate. Prey consumption by the salamanders was monitored for 10 d, and the total number of flies consumed over this 10-day-period was determined. Fruit fly consumption was measured as the total number of flies eaten by salamanders during the 10-day-period in which we monitored fruit fly consumption.

We examined the effect of salamander sex (male: N = 30; female: N = 39) and nocturnal light levels (0.0001 lx, 0.01 lx, 1 lx, and 100 lx; N = 18, 15, 18, and 18, respectively) on total number of fruit flies consumed by salamanders over a 10-day-period using ANCOVA (IBM SPSS V 28). In this analysis, we used the SVL (mm) and body condition (residuals of remeasured empty-stomach mass, in g, regressed on SVL, in mm) measured at 90 d as covariates. We used two-tailed tests with α = 0.05.

### 2.5. Behavior of Fruit Fly Prey

Salamanders use movement of prey as a visual cue during foraging [64]. If flies in different lighting treatments had different levels of activity, those differences might affect the likelihood of salamanders detecting prey and feeding and could, therefore, alter total number of flies eaten by salamanders in the different lighting treatments. To determine whether fruit fly movement at night may have affected the foraging of the salamanders, we monitored (via IR cameras) the nocturnal locomotor activity of individual fruit flies, *D. virilis.* Nocturnal movements of adult fruit flies were recorded using infrared video and infrared LED lighting (>850 nm) under the same night lighting treatments (0.001 lx, 0.01 lx, 1 lx, and 100 lx) and in the same chambers used in the tail regeneration and fly consumption studies previously described. The fruit fly circadian system is insensitive to infrared light and should not be affected by our video recording protocol [74,75]. Fly movement data (distance moved in 1-h intervals) were extracted from video files using computer tracking (Ethovision 7, Noldus Information Technology, Wageningen, the Netherlands). Flies were individually placed into 9 × 1.5 cm Petri dishes lined with moistened, white filter paper to ensure sufficient contrast for automated tracking of fly movements. Flies were placed into the chambers mid-photophase (1200 h, 100 lx) and allowed to habituate until tracking began.

Fly movements were recorded continuously in each chamber from 1600 h (2 h before lights off) until 0800 h (2 h after lights went on) using 16 monochrome, infrared (IR) black/white video cameras (PC6EX3, SuperCircuits, Austin, TX, USA). Each camera was mounted directly above the Petri dish and fitted with long-wavelength pass filters to block wavelengths <850 nm to ensure that all recordings had the same image quality (using IR illumination only) regardless of visible light intensity in the different lighting treatments. Input from the cameras was sampled at a rate of 1 frame/s to reduce storage space needed and processor load using a GV600 16 camera DVR (digital video recorder; GeoVision, Hong Kong, China) board mounted in a computer. Recordings saved using the GeoMPEG4 codec were exported as AVI files and accelerated 16× (to reduce processing time, without sacrificing tracking accuracy) and saved to AVI format using Windows Media Player. AVI files were uploaded into Ethovision 7.0 for fly movement tracking. In video imagery, flies appeared dark gray, moving on a light gray background; the most effective setting in Ethovision for machine fly detection during tracking was using the differencing method. Size calibrations for images were made before setting tracking parameters, which were individually adjusted and calibrated for each file to maximize tracking accuracy and eliminate subtle differences associated with cameras, alignment, or parallax. Ethovision then tracked fly movements within the Petri dish and generated a data set of total fly movement (cm) in 1-h intervals for each fly. After tracking, each file was replayed along with a cartesian overlay of tracked fly movements to detect any errors in tracking (false or untracked movements), and adjustments to tracking parameters and re-tracking were performed as needed.

## 3. Results

### 3.1. Growth and Tail Regeneration in Red-Backed Salamanders

Over a 90-day-period, salamanders did not grow substantially in body size; the mean ± 1 SD change in SVL (percent) was 0.06 ± 1.63%. However, over a 90-day-period, there was a substantial change in regenerated length of autotomized tails; the mean ± 1 SD change in tail length (percent) from autotomized length to final length was 303.84 ± 73.29%. The tails regenerated to 66.83 ± 9.02% (mean ± 1 SD) of the original tail lengths (prior to autotomy) in 90 d.

**Figure 2:**
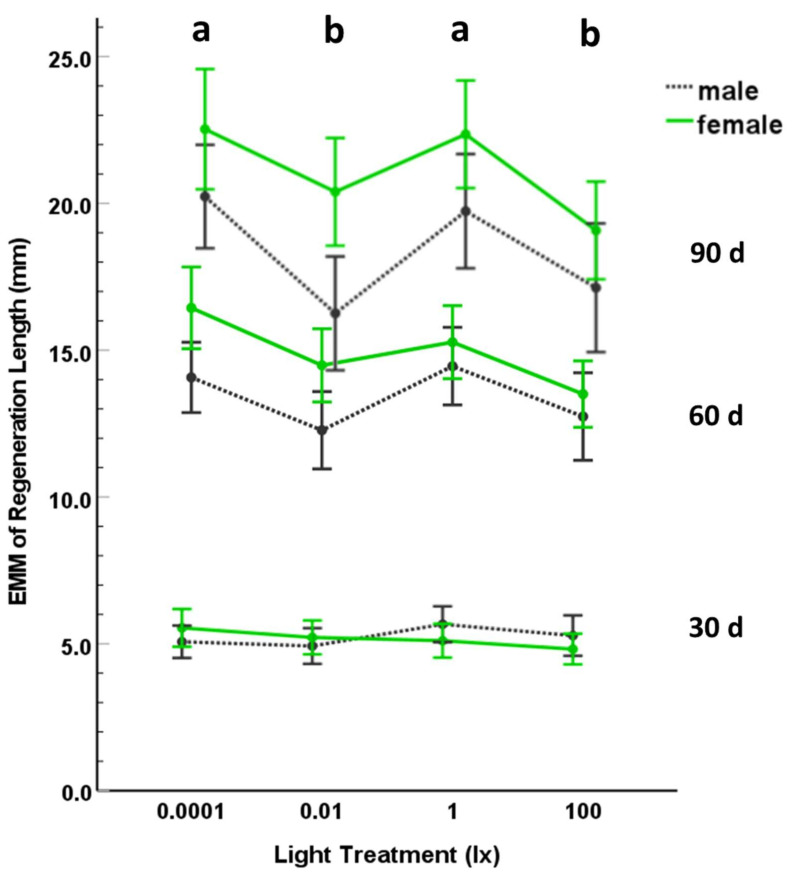
Estimated marginal means (EMM) of regeneration length (mm) for male and female salamanders after 30, 60, or 90 d exposure to nocturnal ambient lighting of 0.0001, 0.01, 1, or 100 lx. The error bars represent 95% CI. Different letters represent treatments that were significantly different.

The variables were normally distributed (using Shapiro–Wilk tests) for light treatment and sex at 30, 60, and 90 d. Additionally, the covariance matrices were not different across treatment groups (*p* = 0.957). We found that the covariate, initial body condition, was not a significant predictor of the regeneration variable (*p* = 0.119; Table 1). We kept the covariate in the analysis because it was a significant predictor of regeneration length at 30 d (*p* = 0.035) and marginal at 60 d (*p* = 0.056); removing the covariate did not change any of the conclusions for the analyses. Using MANCOVA, we found a significant effect of nocturnal light treatment (*p* = 0.021; Table 1; Figure 2) and sex (*p* < 0.001; Table 1) on tail regeneration length, but this effect was only for regeneration lengths at 60 and 90 d (ANCOVA; Table 1). There was no significant interaction effect between light treatment and sex (*p* = 0.298; Table 1). Using post-hoc LSD tests, tail regeneration was significantly less in the 0.01 lx and 100 lx treatments compared to the control dark treatment (0.0001 lx) or the 0.01 lx treatment, but we did not find a significant difference in tail regeneration at 60 or 90 d between the 0.0001 lx and 1 lx treatments or between the 0.01 lx and 100 lx treatments (Table 1; Figure 2). Thus, tail regeneration did not exhibit a simple dose response based on ordered intensity of nocturnal light levels (Figure 2). At 60 and 90 d, tail regeneration length was greater for females than for males (Table 1). This difference between males and females was not attributable to a difference in initial body condition as there was no significant difference in initial body condition (independent *t*-test: *t* = 1.755; df = 74; *p* = 0.083) between males ($\bar{x}$ ± 1 SD = 0.32 ± 1.44) and females ($\bar{x}$ ± 1 SD = −0.29 ± 1.58). However, males and females did differ in initial SVL (independent *t*-test: *t* = −2.577; df = 74; *p* = 0.012; male $\bar{x}$ ± 1 SD = 40.84 ± 2.58; female $\bar{x}$ ± 1 SD = 42.57 ± 3.19), which may account for variation in regeneration rates between the sexes [72].

### 3.2. Consumption of Fruit Fly Prey

**Figure 3:**
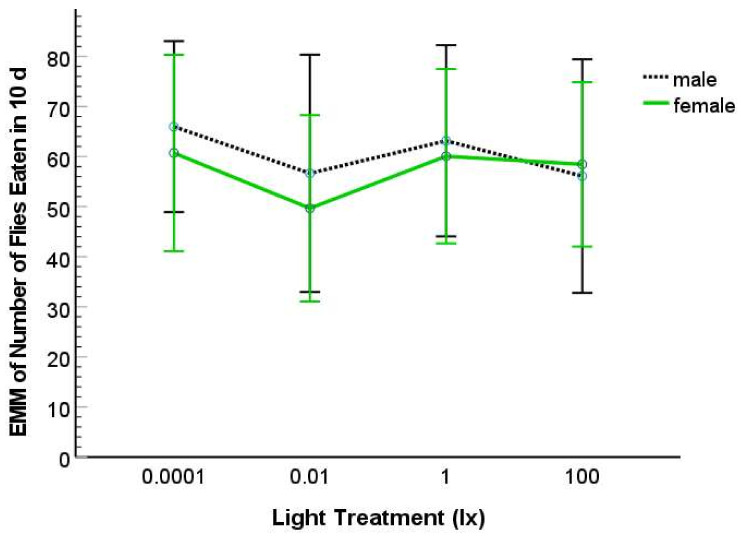
Estimated marginal means (EMM) for number of flies that male and female salamanders consumed in a 10-day-period when exposed to 0.0001, 0.01, 1, or 100 lx ambient nocturnal lighting. The error bars represent 95% CI.

The variable, total number of flies consumed, was normally distributed for each light treatment and sex using Shapiro–Wilk tests (with *p* > 0.05) but did not exhibit equality of error variance (Levene’s Test: *p* < 0.001). Using ANCOVA, we found that both SVL (Type III SS = 10394; F = 14.491; df = 1; 59; *p* < 0.001) and body condition (Type III SS = 12379; F = 17.258; df = 1; 59; *p* < 0.001) were significant as covariates. We found no significant effect of nocturnal light treatment (Type III SS = 870; F = 0.404; df = 3; 59; *p* = 0.751), sex (Type III SS = 129; F = 0.180; df = 1; 59; *p* = 0.673), or interaction effect of light treatment by sex (Type III SS = 185; F = 0.086; df = 3 59; *p* = 0.967) on fly consumption (Figure 3). The same non-significant effects of light and sex were found if we removed one or both the covariates from the analysis.

### 3.3. Movement of Fruit Flies

**Figure 4:**
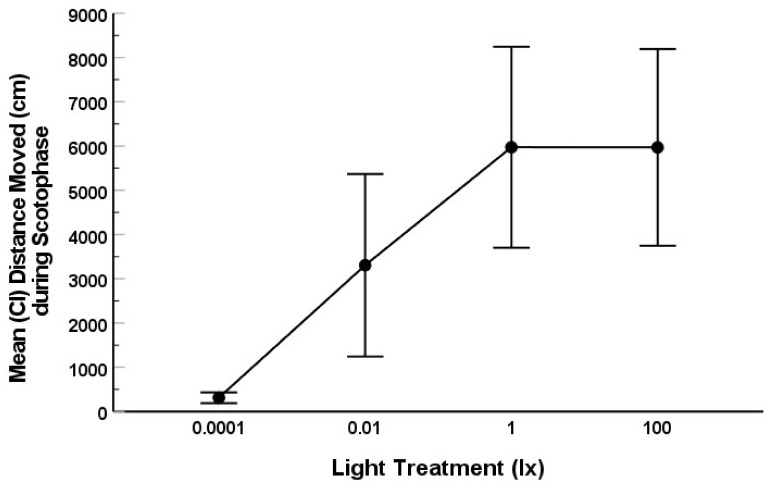
Mean (and CI) total distance moved (cm) by fruit flies from the time lights went off (clock hour 1800 h) until lights on (clock hour 0600 h) for each lighting treatment: 0.0001, 0.01, 1, or 100 lx ambient nocturnal lighting. The error bars represent 95% CI. Different letters represent treatments that were significantly different.

**Figure 5:**
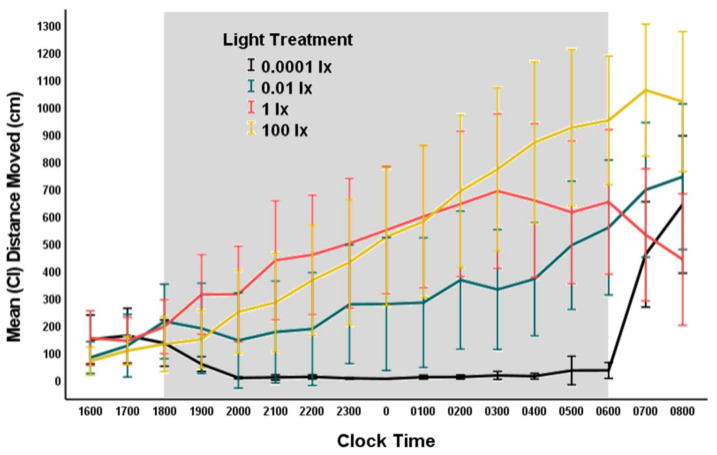
Movement of fruit flies in each nocturnal light treatment (0.0001, 0.01, 1, and 100 lx) at 1-h-intervals starting 2 h before lights off (1800 h) until 2 h after light on (0600 h). Time on x-axis represent the starting time of the 1-h-interval. The error bars represent 95% CI.

Movement of fruit flies (total distance moved in cm) during scotophase was significantly affected by nocturnal illumination (Type III SS = 510316794; ANOVA: F = 8.356; df = 3; 91; *p* < 0.001). The variance among lighting treatments for movement was not equal (Levene’s test: *p* < 0.001), as flies in the 0.0001 lx (dark control) treatment exhibited very little movement overall (Figure 4 and Figure 5). Movement of fruit flies nearly exhibited a dose response: those exposed to the darkest illumination (0.0001 lx) moved significantly less during scotophase than those in the 0.01 (*p* = 0.025), 1 (*p* < 0.001), or 100 lx (*p* < 0.001) treatments (Figure 4). Flies in the 0.01 lx treatment moved significantly less during scotophase than those in the 1 (*p* = 0.025) and 100 lx (*p* < 0.044) treatments (Figure 4). However, the movement of flies in the 1 and 100 lx treatments did not differ statistically (*p* = 0.998).

Movement of flies in the darkest light treatment (dark control, natural nocturnal lighting) was minimal, whereas, for those in the lighted treatments, activity increased throughout the night, with more activity during the early morning hours (0300–0600 h) before the switch to day lighting (Figure 5). We used a repeated-measures ANOVA with a Greenhaus–Geisser correction (0.614) because the assumption of sphericity was not met (Mauchly’s test: *p* < 0.001) to examine the movement behavior of flies over several time periods, including 1800 (lights off), 2100, 0000, 0300, and 0600 h (lights on). We found a significant effect of time of night (Type III SS = 9328857; F = 20.676; df = 2.46,223.48; *p* < 0.001), nocturnal light treatment (Type III SS = 18125507; F = 10.050; df = 3,91; *p* < 0.001), and an interaction effect of time and light treatment (Type III SS = 7608514; F = 5.621; df = 7.37,223.48; *p* < 0.001) on movement of fruit flies. Post-hoc analyses using ANOVA at each time interval indicated no significant differences in movement among light treatments when lights switched to night lighting (1800 h), but significant differences among treatment groups occurred at other time intervals (Table 2 and Figure 5).

## 4. Discussion

We found that even very small amounts of ALAN significantly affected tail regeneration in the eastern red-backed salamander, *P. cinereus*. However, the effect of ALAN on tail regeneration was not monotonic but much more complex; regeneration was highest in the dark control (0.0001 lx) and 1 lx treatments and significantly lower in the 0.01 and 100 lx light treatments (Figure 2). These data are inconsistent with our hypothesis that the effect of nocturnal illumination on tail regeneration would be a dose-dependent response with either increasing or decreasing rates of tail regeneration with increasing nocturnal illumination, suggesting a more complex physiological or behavioral response to ALAN. The tail has multiple functions in *P. cinereus* as well as other salamanders, including energy storage [34,36,37,76,77], signaling and territorial defense [38,39,40,41], and antipredator behavior [33,34,78,79], and the ability to regenerate the tail quickly may impact survival and reproductive success [34,35]. An important function of the tail is its use as an escape mechanism when the salamander is attacked by a predator and the tail is autotomized and produces noxious and adhesive secretions that deter and increase handling time for predators [33,34,78,79]. The tail is also important for fat storage during winter when salamanders do not feed; for example, salamanders at higher elevations sequester more fat than salamanders at lower elevations [36]. Fat storage also seems important for reproductive success and oocyte maturation [77] and brooding females that have intact tails produce more ova the following year than females that have lost their tails [37]. Tail loss also impacts territorial behavior including scent marking (rate of deposition of pheromones via post-cloacal press [38] and aggressive displays [39,40], such that salamanders with shortened tails are at a disadvantage during territorial contests and likely subjected to increased aggression from opponents [41]. Presumably, because of the importance of having a complete tail, regeneration and elongation of the tail occurs even in salamanders that are not fed for extended periods of time (>9 mo) [35]. Thus, ALAN-induced differences in tail regeneration are expected to have consequences for the population ecology of these territorial salamanders as well as other species of salamanders with similar life histories.

Although ALAN altered tail regeneration rates in salamanders with slower rates of regeneration at nocturnal illuminations of 0.01 and 100 lx, this effect was unlikely the result of differences in activity of the fruit fly prey. We did find that the activity of fruit flies (*D. virilis*) was profoundly affected by ALAN, with overall nocturnal movement increasing as nocturnal illuminations increased. This result was most obvious at lower levels of ALAN (dALAN) with flies significantly more active in the 0.01 lx treatment than in the dark control and with flies even more active in the 1 and 100 lx treatments than in the 0.0001 and 0.01 lx treatments (Figure 4). It should be noted that our brightest night lighting treatment was 100 lx, which is equivalent to dim room lighting, typical of what might be experienced under the forest canopy on an overcast day, and not nearly as bright as unobscured day lighting (sunlight). Studies in another species of fruit fly, *D. (S.) melanogaster,* found an increase in activity when flies were exposed to dALAN (0.03 lx) [60] as opposed to virtual darkness as well as avoidance of activity in brightly lit areas (100 lx or greater) [63]. We saw similar patterns of activity in *D. virilis*, with significantly more activity in dALAN (0.01 lx) than in the dark control treatment (0.0001 lx). We also found increased activity in the 1 lx treatment under our lighting regimen, similar to *D. (S.) melanogaster* (with most activity at 7.5 lx regardless of time of day) [63]. However, our data differed from [63] in that we found that activity was similar in both the 1 lx and in the brighter 100 lx treatment, which may be due to species–specific variation in rhythmic behavior or differences in experimental design.

Timing of fly activity is controlled, in part, by the circadian system [80]. Research has demonstrated that a nocturnal illumination of 0.03 lx was sufficient to shift the ‘temporal niche’ of *D. (S.) melanogaster* to becoming active nocturnally rather than being crepuscular [60]. The flies in that study appeared to have a dim light preference and did not simply become active for more hours a day but shifted their initiation of activity from the morning period (M) to the evening period (E). In another species of fruit fly, *Drosophila* (*Sophophora*) *biarmipes*, dim light (0.03 lx) at night sped up re-entraintment (recovery of normal circadian pattern) after rhythm disruption more effectively than did total darkness [81]. Similar effects of dim lighting have been observed in Syrian hamsters (*Mesocricetus auratus*), where nocturnal illumination of 0.004 lx (equivalent to dim moonlight) was sufficient to accelerate re-entrainment relative to total darkness after disruption of circadian cycles [82], demonstrating that even small amounts of light during scotophase can alter periodic locomotor rhythms, when compared to what occurs in studies that employ total darkness as a control treatment. It has been suggested that such an effect might be caused by dim lighting disrupting the coupling between the M and E oscillators [81], though data for mice (*Mus musculus*) at least indicate that this effect is mediated by clock genes outside the SCN [83]. Regardless of the mechanism, dALAN is able to alter periodic locomotor behavior in multiple species of fruit flies and in mammals. We did not include a total darkness control treatment opting instead for what we perceived to be a more natural 0.0001 lx dark control equivalent to starlight. Unless animals are fossorial, they are unlikely to encounter total darkness above ground at night [84] and, therefore, total darkness may not be the most effective control lighting treatment in studies of organisms that do not regularly experience total darkness at night. Without the total darkness control we are unable to assess how well or poorly fly activity in our dark control might match that of total darkness in other studies. What is clear from our study, however, is that even small increases in dim lighting can alter fly nocturnal behavior.

Salamanders provided prey (*D. virilis*) with different activity levels (amount of movement) at different nocturnal illuminations may experience different rates of prey encounter and/or detection and these differences could potentially affect prey consumption rates and, ultimately, tail regeneration rates; however, that is not what we found. Tail regeneration was not detectably changed by prey consumption because we saw no significant differences in prey capture and consumption by salamanders in different nocturnal lighting treatments, when examined for a 10-day-period. Additionally, food consumption does not seem to alter the regeneration rate in *P. cinereus* monotonically. One study found that length of tail regeneration was not statistically different in individuals of *P. cinereus* given low or high quantities of prey items (*D. melanogaster*), rather quantity of food affected the volume of fat in the regenerated tails [35], which we did not assess in our study. Thus, in spite of substantial differences in fly activity in the different lighting treatments, consumption of fruit fly prey was most likely not a factor influencing tail regeneration because (1) salamanders ate similar quantities of prey in our experiment (Figure 3), (2) regeneration lengths are not impacted by food consumption in this species of salamander [35], and (3) fruit fly activity did not vary in the same way as tail regeneration among light treatments (Figure 2 and Figure 4). We speculate that the complex, non-linear response of regeneration to different amounts of dALAN seen in our experiment is not due to prey behavior but rather is due to (1) physiological factors that are under hormonal (e.g., melatonin, serotonin, prolactin, and corticosterone) control or (2) genetic control where different sets of genes may be active at different scotopic illuminations and that their products may cause differences in endocrine responses under different levels of dALAN.

ALAN can, in some species, result in increased levels of glucocorticoid hormones, such as corticosterone, through the hypothalamic–pituitary–adrenal (HPA) axis [85] (the HPA/intrarenal (I) axis in amphibians [86]). For example, corticosterone levels were higher in tadpoles reared in ALAN treatments compared to dark controls [53]. Additionally, *P. cinereus* is nocturnally active on the forest floor (Figure 1 in [59]) and negatively phototactic, avoiding bright light (e.g., 1400 lx), presumably as a mechanism to avoid high temperatures, desiccation, or predation, although light avoidance occurs even under controlled conditions of temperature and humidity [58]. Thus, under higher illuminations (i.e., constant 100 lx), salamanders may exhibit stress-related responses, including increases in corticosterone, such as that demonstrated in frog tadpoles after a 14-day-exposure to ALAN (constant light at 250 lx) [53]. This is of particular significance because corticosterone can impede regeneration. In another plethodontid salamander, *D. ochrophaeus,* exogenous corticosterone delivered via skin patches slowed wound healing [87] and tail regeneration [53]. If ALAN results in a chronic increase in corticosterone, we would predict slower regeneration rates at higher light levels. Such a stress response could explain our lower rates of regeneration occurring at 0.01 and 100 lx, but would not be consistent with the higher rate of regeneration in the 1 lx treatment that had similar regeneration rates as the dark control. However, evidence summarized in [86] suggests that in at least some species of plethodontid salamanders, elevated corticosterone levels may not be associated with chronic stress; although the stressors they investigated did not include ALAN. Species–specific modulation of a corticosterone response to ALAN (e.g., downregulation) can occur, as was found for tadpoles exposed to pulsed ALAN [53]. Additional research examining corticosterone levels in plethodontid salamanders under various ALAN conditions is important for understanding the role of ALAN in influencing corticosterone levels and tail regeneration in this species. This is an area with little research and is an important topic in understanding factors influencing tissue regeneration [88].

Salamanders, as all vertebrates, show peak levels of melatonin during naturally dark periods and peak serotonin levels during photophase [45,89]. The pineal gland converts serotonin to melatonin during dark periods and production and secretion of melatonin is suppressed during daylight hours, resulting in higher levels of melatonin at night under natural lighting conditions. Although in non-mammalian vertebrates melatonin can be synthesized by the eyes and other organs [13,85], melatonin produced in the eyes does not seem to be as important as pineal melatonin in tissue regeneration in at least one species of nocturnal lizard [42]. The natural light:dark cycles have resulted in a conserved and crucial role for the production of melatonin in regulating many physiological functions, especially those associated with daily and seasonal rhythms [13,85,90,91,92]. ALAN, even at low levels, can disrupt the melatonin cycle by suppressing melatonin production at night [13,45,87]. While no studies have directly investigated the effect of melatonin on tail regeneration in salamanders, studies on limb regeneration in newts provides some evidence for the role of melatonin in salamander tissue regeneration. Eastern red-spotted newts (*N. viridescens*) kept under constant light at night regenerated lost limbs faster than those maintained in complete darkness [43]. Additionally, newts (*N. viridescens*) with blocked pineal glands regenerated limbs more slowly than animals with normal, exposed pineal glands, providing evidence of the importance of the melatonin cycle in tissue regeneration [44]. Research on tail regeneration in the nocturnal gekkonid lizard, *H. flaviviridis* provides similar results. In a controlled experiment, tail regeneration was greatest in lizards kept under bright continuous daylight of either 2500 or 638 lx, compared to a 12L:12D photoperiod, and was slowest in continuous dark [42]. Pinealectomized lizards exhibited reduced growth no matter the lighting treatment compared to a sham control [42], and in another experiment [93], exogenous melatonin enhanced regeneration rates in lizards on a natural photoperiod when given at 1700 h and suppressed regeneration when administered at 0700 h, indicating that the natural, nocturnal production of melatonin is important in tail regeneration.

Rhythmicity of melatonin and serotonin secretion may influence regeneration by modulating the secretion of prolactin [88]. Studies have shown that prolactin significantly increases regeneration in newt forelimbs and newt and lizard tails [51,52,94,95]. Thus, an increase in regeneration occurring under conditions of constant light may be due to high levels of serotonin, which stimulates the release of prolactin [44,88]. Interestingly, pinealetomized lizards given exogenous prolactin regenerated tails faster than those not given prolactin but did not regenerate tails as much as intact lizards receiving prolactin, suggesting that prolactin stimulates tail regeneration but that the cyclical production of melatonin is also important in the process [52]. In our experiment, we did not find support for constant light increasing tail regeneration rates. At constant light levels of 100 lx (substantially lower than day-lighting illuminations used in other experiments we discussed), we found reduced tail regeneration lengths compared to tail lengths of salamanders exposed to a normal dark night (0.0001 lx). However, at intermediate levels of ALAN, we did find more regeneration at 1 lx than 100 lx. Because of the complex relationship between internal and external factors that may influence regeneration in different ways within and among species [88], constant dALAN may result in a more complex response to light levels in our salamanders than can be predicted by individual factors.

Dim ALAN, at particular illuminations, may simulate conditions of dawn or dusk and may differentially regulate the expression of genes associated with the morning (M) or evening (E) oscillators. Under natural conditions, illuminations increase gradually at the end of scotophase triggering regulation of the genes associated with the morning (M) oscillator. If expression of different genes is regulated by different environmental illuminations, then different genes will be expressed as light levels increase at dawn or decrease at dusk. ALAN illuminations of 1 lx and below have been recorded in natural habitats [11,59]. Salamanders exposed to 1 lx in our experiment were effectively temporally trapped in perpetual twilight, greatly increasing the duration of subjective morning (M) or evening (E) conditions. Under normal, clear-sky, natural conditions, twilight illuminations of 1 lx at dawn and dusk only happen for a few minutes each day (though they may happen under dense forest canopy during the day under heavy cloud cover). If nocturnal illuminations are held at 1 lx for the entirety of scotophase, then the normal pattern of gene expression at twilight would be maintained constantly through the night and salamanders under those conditions would never experience gene expression typical of normal scotopic conditions. This same potential problem also exists at 0.01 lx (full moonlight) but organisms are likely to experience full moon lighting for longer periods during months when the full moon is present above the horizon during scotophase.

Additional studies addressing the physiological mechanisms responsible for variation in rates of regeneration that we have observed at ecologically-relevant levels of light pollution would greatly benefit our understanding of how regeneration rates are influenced by ALAN. The most studied models for tissue regeneration in vertebrates are lizards and newts (*N. viridescens*). Adult eastern red-spotted newts are aquatic and lizards have epidermal scales for reducing desiccation, whereas *P. cinereus* is terrestrial and thin-skinned. Thus, *P. cinereus* is much more likely than lizards and newts to be impacted by stress associated with water loss [86] that may influence its response, especially a stress response, to lighted conditions above ground. Additionally, no other studies of terrestrial salamanders have been published examining the effect of ALAN at ecologically relevant levels; therefore, it is unknown if the complex, nonlinear pattern seen in our tail regeneration data can be generalized to other species or whether it might be unique to this system.

How likely is it that eastern red-backed salamanders, or fruit flies for that matter, might encounter these levels of ALAN in their natural habitats? Direct glare from streetlamps along roadways, edificarian lighting, such as security lighting, or other sources does not stop when it hits the forest edge. Light from such sources can travel great distances, though it diminishes with distance as the inverse of the squared distance. Skyglow from cities, industrial sites, and sports or other large illuminated complexes occurs when light from those sources is reflected off moisture in the atmosphere back to the ground far from its sources. We have measured combined contributions of glare and sky glow in a protected wetland (Utica Marsh adjacent to Utica, NY) of 0.1–1.0 lx across large areas of that wetland, which contains many species of amphibians and insects. It seems that 23% of the land surface of the Earth (between 75 degrees N and 60 degrees S) is seriously light-polluted and that 46.9% of the United States and 88.4% of the European Union land area experience significant levels of sky glow [1]. Overlaying the Artificial Night Sky Brightness Atlas [1] for the Northeastern United States onto the range map for *P. cinereus* [96] demonstrates that almost the entire range of the species experiences sky glow on a nightly basis. Such pervasive dALAN, which may widely affect ecologically important nocturnal species, such as *P. cinereus*, raises significant conservation concerns.

## 5. Conclusions

Very small amounts of dALAN that is pervasive across much of the range of *P. cinereus* caused a complex, non-monotonic effect on tail regeneration rates in the eastern red-backed salamander, *P. cinereus*, that most likely involves the interaction of several hormones and metabolic processes. ALAN also altered the activity of fruit fly prey (monotonic response) but not in a way that obviously impacted food consumption and tail regeneration in salamanders. Thus, dALAN has the ability to affect tail regrowth, which can in turn affect the survival and reproduction of this important species of salamander.

## Figures and Tables

**Table 1:** MANCOVA analyzing the effect of nocturnal ambient light (light treatment, lx), sex, and the interaction of light and sex on regeneration lengths of tails (mm) at 30, 60, and 90 d after inducing tail autotomy in red-backed salamanders. Initial body condition (residuals of mass on snout-vent length, SVL) was used as a covariate in the analysis. Significant multivariate tests were further analyzed with ANCOVA. Significant ANCOVA tests for light treatment were further analyzed with LSD tests to determine pairwise differences.

Source of Variation	Wilks’ λ	Type III SS	F	df	*p*
Initial Condition	0.915		2.024	3, 65	0.119
Light Treatment	0.746		2.251	9, 158.3	0.021 *
Regeneration, 30 d		1.559	0.636	3, 67	0.595
Regeneration, 60 d		62.064	5.369	3, 67	0.002 *
0.0001 vs. 0.01 lx					0.005 *
0.0001 vs. 1 lx					0.543
0.0001 vs. 100 lx					0.002 *
0.01 vs. 1 lx					0.024 *
0.01 vs. 100 lx					0.701
1 vs. 100 lx					0.009 *
Regeneration, 90 d		166.285	6.664	3, 67	<0.001 *
0.0001 vs. 0.01 lx					0.002 *
0.0001 vs. 1 lx					0.723
0.0001 vs. 100 lx					0.001 *
0.01 vs. 1 lx					0.006 *
0.01 vs. 100 lx					0.821
1 vs. 100 lx					0.003 *
Sex	0.749		7.248	3, 65	<0.001 *
Regeneration, 30 d		0.071	0.087	1, 67	0.769
Regeneration, 60 d		41.983	10.895	1, 67	0.002 *
Regeneration, 90 d		133.616	16.064	1, 67	<0.001 *
Light Treatment*Sex	0.852		1.200	9, 158.3	0.298

* Significant at α = 0.05.

**Table 2:** Post-hoc comparisons of fruit fly activity (cm moved) using ANOVA at several 1-h-time-intervals.

Source of Variation	Type III SS	F	df	*p*
Clock Time 1800 h	126038	0.657	3, 91	0.581
Clock Time 2100 h	2301081	4.710	3, 91	0.004 *
0.0001 vs. 0.01 lx				0.161
0.0001 vs. 1 lx				<0.001 *
0.0001 vs. 100 lx				0.022 *
0.01 vs. 1 lx				0.027 *
0.01 vs. 100 lx				0.355
1 vs. 100 lx				0.189
Clock Time 0000 h	4554840	6.046	3, 91	<0.001 *
0.0001 vs. 0.01 lx				0.064
0.0001 vs. 1 lx				<0.001 *
0.0001 vs. 100 lx				<0.001 *
0.01 vs. 1 lx				0.064
0.01 vs. 100 lx				0.701
1 vs. 100 lx				0.092
Clock Time 0300 h	8550782	9.243	3, 91	<0.001 *
0.0001 vs. 0.01 lx				0.055
0.0001 vs. 1 lx				<0.001 *
0.0001 vs. 100 lx				<0.001 *
0.01 vs. 1 lx				0.027 *
0.01 vs. 100 lx				0.007 *
1 vs. 100 lx				0.620
Clock Time 0600 h	10201286	12.782	3, 91	<0.001 *
0.0001 vs. 0.01 lx				<0.001 *
0.0001 vs. 1 lx				<0.001 *
0.0001 vs. 100 lx				<0.001 *
0.01 vs. 1 lx				0.531
0.01 vs. 100 lx				0.010 *
1 vs. 100 lx				0.048 *

* Significant at α = 0.05.

## Data Availability

The data presented in this study are available upon request.
